# Antibody response against three *Plasmodium falciparum* merozoite antigens in Mamuju District, West Sulawesi Province, Indonesia

**DOI:** 10.1186/1475-2875-13-381

**Published:** 2014-09-25

**Authors:** Nurhayana Sennang, Stephen Rogerson, Sitti Wahyuni, Irawan Yusuf, Din Syafruddin

**Affiliations:** Department of Clinical Pathology, Faculty of Medicine, Hasanuddin University, Makassar, 90245 South Sulawesi, Indonesia; Department of Medicine, Post Office Royal Melbourne Hospital, University of Melbourne, Melbourne, Victoria Australia; Department of Parasitology, Faculty of Medicine, Hasanuddin University, Makassar, 90245 South Sulawesi, Indonesia; Department of Physiology, all at Faculty of Medicine, Hasanuddin University, Makassar, 90245 South Sulawesi, Indonesia; Eijkman Institute for Molecular Biology, Jakarta, 10430 Indonesia

**Keywords:** Antibody responses, Merozoites antigens, MSP2, EBA175 and PfRh2a, Indonesia

## Abstract

**Background:**

Malaria endemicity in the archipelago of Indonesia varies substantially across regions. Following the government’s plan for a malaria elimination programme in Indonesia, baseline malaria surveys were conducted in Mamuju District, West Sulawesi Province, Indonesia to re-assess the malaria situation prior to the establishment of an evidence-based malaria elimination programme in the area. The present study aims to determine the antibody response to three merozoite antigens among the inhabitants of the district.

**Methods:**

Antibodies were measured following elution from filter-paper blood spots collected during cross-sectional surveys in the dry and wet season in 2010. Enzyme-linked immunosorbent assays using three merozoite antigens, MSP2, EBA175 and PfRh2a were conducted. A positivity threshold was determined by samples from unexposed individuals and the difference in antibody level against each antigen and correlation of antibody level in different age groups and seasons were statistically analysed.

**Results:**

A total of 497 subjects, 248 in dry and 249 in wet season, aged between 0.6 and 78 years were included. The prevalence of positive antibody responses to MSP2, EBA175 and PfRh2a antigens in dry season were 27.82, 27.42 and 25.81%, respectively. In wet season, the antibody prevalences were 64.26, 64.66 and 61.45%. The antibody levels to the three antigens were all higher in older age groups and also significantly higher in the wet season. The antibody levels also correlated positively with the *Plasmodium falciparum* infection status of the subjects.

**Conclusion:**

MSP2, EBA175 and PfRh2a induce antibody responses among the subjects in Mamuju District, and the prevalence is significantly higher in wet season. The level of antibody also correlates significantly with age and malaria positivity. The overall results indicate the antigens might be used as a target for vaccines against *P. falciparum* infection and as markers for malaria exposure.

## Background

Malaria remains one of the major public health problems in many tropical countries around the world, including Indonesia. In 2013, of the 97 countries with ongoing malaria transmission, 11 were in pre-elimination phase and seven in elimination phase [1]. In Indonesia, malaria endemicity varies substantially across different regions, in which Java and Bali islands are very low endemicity, whereas outer islands are meso- to highly endemic. The government of Indonesia developed a plan for malaria elimination in 2009 and each region has set different timelines for elimination phases [2]. In eastern Indonesia, malaria control efforts continue to rely heavily on chemotherapy and vector control through provision of insecticide-treated nets. As in many other countries in the Asia-Pacific region, *Plasmodium falciparum* and *Plasmodium vivax* are the major causative species of malaria in Indonesia. *Plasmodium malariae* and *Plasmodium ovale* occurred infrequently, mainly in eastern Indonesia.

Malaria symptoms in humans are exclusively caused by the erythrocytic stage of the parasite and therefore vaccine development is mainly focused to prevent the invasion and development of the parasite in this stage. Children and pregnant women are the most vulnerable groups in areas of stable malaria, and after repeated exposures individuals develop immunity to the blood stage parasite, thereby reducing the risk of clinical symptoms and life-threatening complications. To date, various merozoite proteins have been implicated in the erythrocyte invasion, and some of these have been identified as targets for vaccine development and markers for epidemiologic studies [3]. Antibodies to merozoite antigens are considered important targets of protective antibodies and are thought to function *in vivo* by inhibiting merozoite invasion of erythrocytes, opsonizing merozoites for phagocytosis, and inducing antibody-dependent cellular inhibition.

Invasion of the erythrocytes by the malarial parasite involves several families of merozoite proteins during the initial step of attachment, reorientation, penetration, and formation of the parasitophorous vacuole. This includes merozoite surface protein family (MSP), erythrocyte binding-like protein (EBP) and the reticulocyte binding-like or reticulocyte homologue proteins (RBL or PfRh). Many of the proteins are being evaluated as targets for anti-malarial vaccine candidates [4, 5]. Merozoite surface protein 2 (MSP2) is one of the MSP family members that has been extensively studied and the antibody response to this protein has been associated with protection against malaria. Erythrocyte binding antigen-175 (EBA-175) is a 175-kilodalton EBP of *P. falciparum* parasites that mediates erythrocyte invasion. EBA-175 is found in the micronemes of merozoites, which secrete EBA-175 to bind erythrocytes that are ready to be invaded [6, 7]. Analysis of sera from malaria-endemic areas to determine the presence of specific antibody EBA-175 revealed some relationships with protection in children with higher antibody titres [8].

Reticulocyte binding-like protein (protein RBL) of *Plasmodium* is one of the classes of type 1 transmembrane ligand parasites localized in the rhoptry. A recent study indicated that antibodies that target the binding domain of PfRh2a inhibited the invasion of the erythrocyte by the merozoites [9].

The present study aims to measure antibody responses against merozoite proteins MSP2, EBA-175 and PfRh2a in Mamuju District, West Sulawesi, a malaria-endemic region in eastern Indonesia and to evaluate the association between antibody levels, malaria infection, age, and season.

## Methods

### Study site

**Figure 1:**
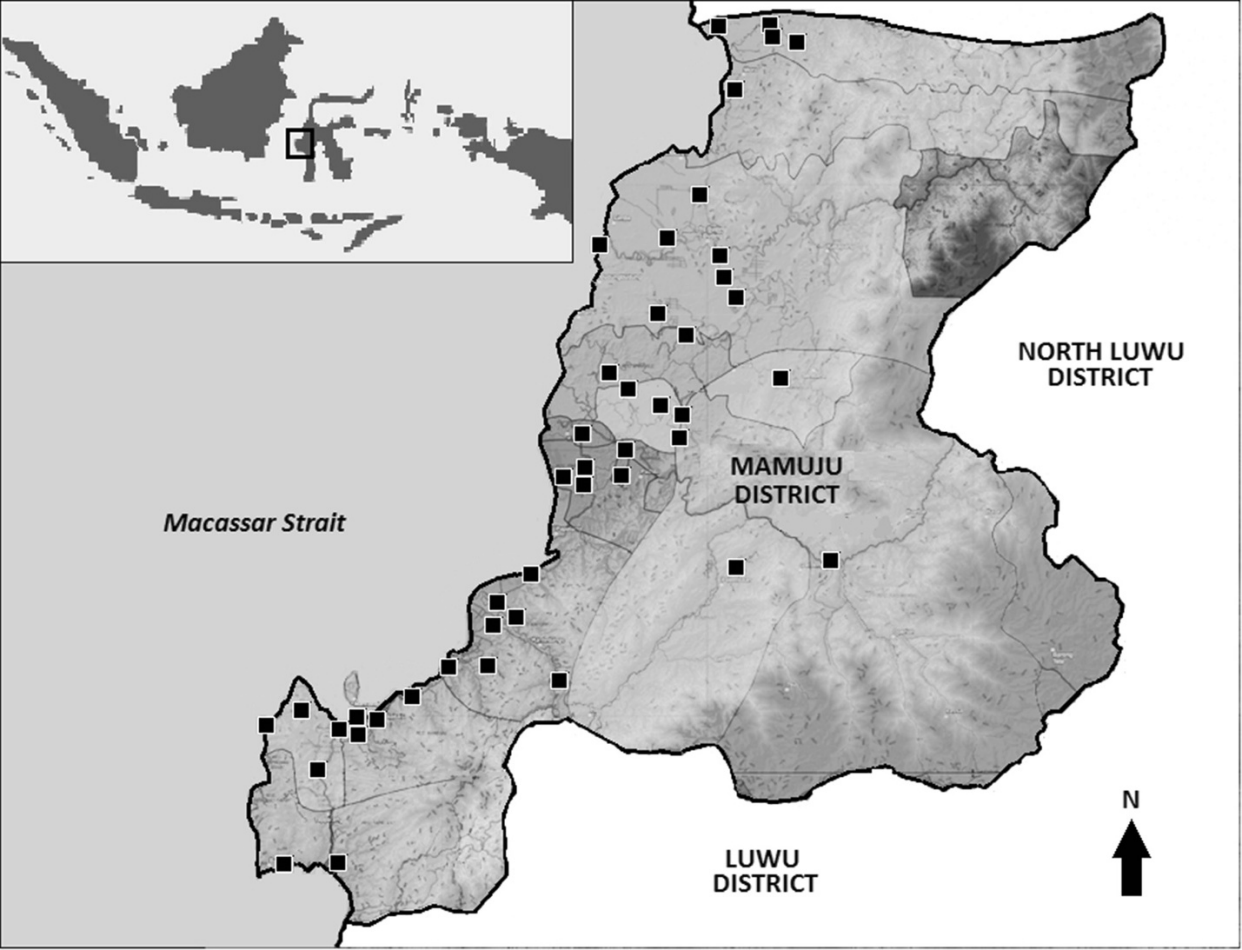
**Mamuju District in West Sulawesi indicated by the box and location within the Indonesian archipelago (inset).** Not to scale.

The study was a part of research activities to establish an evidence-based malaria control programme in the Mamuju District, West Sulawesi Province, Indonesia through assessment of the baseline malaria prevalence in the area. Mamuju District is located within 2°40’- 3°39’ south latitude and 118°46’- 119°25’ east longitude and divided into 15 subdistricts. The District occupies an area of 8,014 sq km along the midwest coastal region of the island of Sulawesi with a total population of 336,973 (Figure 1). The population mainly consists of Mandarese and Buginese ethnic groups with some resettlers from the islands of Java, Bali and Lombok. This area is known to be endemic for malaria and lymphatic filariasis [10]. Multistage, random sampling cross-sectional surveys was conducted in Mamuju District, West Sulawesi Province, Indonesia during the dry and wet seasons of 2010. Subjects were asked to provide written informed consent for finger-prick blood smears and blood blots for DNA and immunologic analysis. This study received ethics approval for the use of human subjects from the Ethics Committee, Faculty of Medicine, Hasanuddin University.

### Sample selection

A total of 4,406 and 4,706 samples in the forms of filter paper blood spots were collected during the baseline malaria surveys performed during the dry (August) and rainy season (December), respectively, in 2010. The samples were further selected for immunological assays using all confirmed falciparum malaria cases by microscopy and polymerase chain reaction amplification (PCR). In addition, 5% of the total negative samples, selected randomly and representing proportionally all the villages’ population, were also included.

### Deoxyribonucleic Acid (DNA) extraction and polymerase chain reaction amplification

The parasite DNA was extracted from the filter paper using Chelex-100 ion exchanger method as described previously [11]. The DNA extract was used as template for the nested PCR using oligos that target the parasite MSP2 genes [12].

### Immunologic assays

#### Elution of antibodies from blood spots

Elution of the antibodies from the filter paper was essentially done as that described previously with slight modification [13, 14]. Plastic bags containing blood spots were allowed to return to ambient temperature before opening. Discs of 4.5 mm in diameter were cut from the filter paper using a paper puncher (Kangaro punch, Kanin India Ltd, Delhi, India). The individual disc was transferred into a 1.5 ml Eppendorf microtube containing 0.05% Tween 20 and 0.5% bovine serum albumin (BSA) in phosphate-buffered saline (PBS). Following incubation for two hours at room temperature (RT) on a shaker and vortexing for a few seconds, the liquid was pipetted into a new, sterile Eppendorp microtubes and kept at −20°C until use.

#### Enzyme-linked immunosorbent assays

Recombinant MSP2, EBA175 and Rh2A9 proteins were all gifts from Prof J Beeson of the Burnet Institute, Australia. Total immunoglobulin (IgG) responses to MSP2 (3D7 allele), EBA175 and Rh2a were measured using enzyme-linked immunosorbent assay (ELISA) as described previously [9, 15]. Briefly, the sera was extracted from the filter paper using a paper puncher with diameter of 6 mm and transfered to a 1.5 ml Eppendorf micotube containing 500 ml PBS, 0.05% Tween 20 and 0.5% BSA. Following incubation on a rotatory shaker at RT for two hours, the supernatant was transferred to a new, sterile 1.5 ml Eppendorf microtube and used for ELISA. Antibody levels were expressed in arbitrary units (AUs), calculated by dividing the optical density (OD) of the sample by the mean OD plus three standard deviations (SD) of samples from control samples (samples from individuals never exposed to malaria). Positive control samples from individuals with known antibodies to these antigens were placed on each plate and values were similar on all test days.

### Statistical analysis

Statistical analysis was performed using software of IBM SPSS Statistics 22. To test for differences in antibody levels for the three antibodies, MSP2, EBA175, and PfRh2a, based on sampling time, malaria positivity and age, Mann–Whitney test was used. The median differences of the three antibodies based on age group were determined using Kruskal Wallis test. Spearman correlation test was used to determine the association between the level of MSP2, EBA175 and PfRh2a antibodies with age.

## Results

### Demographic characteristics of the subjects

Demographic characteristics of the subjects are shown in Table 1. Of the total 497 subjects selected, 207 were men and 290 were women. Fifty-five subjects were < five years old (11.07%). Samples collected in the dry season were 248 (49.90%). There were 59 samples that were positive for *P. falciparum* based on the results of PCR (Table 1).

**Table 1:** **Characteristics of study subjects**

Variable	n	%
Sex		
Male	207	41.65
Female	290	58.35
Age category		
<= 5 years	55	11.07
> 5 years	442	88.93
Sampling time		
Dry season	248	49.9
Rainy season	249	50.1
*P. falciparum* PCR		
Positive	59	11.87
Negative	438	88.13

### Enzyme-linked immunosorbent assay (ELISA)

#### Merozoite surface protein-2 (MSP2) antibodies

The prevalence of positive antibody response to MSP2 antigens among the subjects in the dry and wet season was 27.82% (median 4.47, IQR 2.76-9.24) and 64.26% (median 5.16, IQR 3.52-9.78), respectively (Table 2). The difference in the antibody level against MSP2 between the dry and wet season was statistically significant (p <0.001).

**Table 2:** **IgG level to MSP2, EBA-175 and PfRh2a** ***Plasmodium falciparum*** **based in the dry and wet season**

Variables	Dry season (n = 248)		Wet season (n = 249)		p*
	Antibody prevalence (%)	Median (IQR)	Antibody prevalence (%)	Median (IQR)	
IgG MSP2	27.82%	4.47 (2.76-9.24)	64.26%	5.16 (3.52-9.78)	<0.001
IgG EBA-175	27.42%	9.71 (6.58-16.54)	64.66%	11.49 (8.82-16.78)	<0.001
IgG PfRh2a	25.81%	3.05 (2.51-6.19)	61.45%	4.47 (3.46-6.03)	<0.001

(*) was determined by using Mann–Whitney test.

#### Erythrocyte binding antigen (EBA175) antibodies

Antibody response to EBA-175 antigen is shown in Table 2. The prevalence of positive antibody response to EBA-175 antigen among the subjects in the dry and wet season was 27.42% (median 9.71, IQR6.58-16.54) and 64.66% (median 11.49, IQR 8.82-16.78), respectively. The difference in the antibody response between the dry and wet season was statistically significant (p < 0.001).

#### *Plasmodium falciparum*rhoptry 2a (PfRh2a) antibodies

Antibody response to PfRh2a antigen in the two collected seasons is shown in Table 2. The prevalence of positive antibody response to PfRh2a antigen among the subjects in the dry and wet season was 25.81% (median level 3.05, IQR 2.51-6.19) and 61.45% (median 4.47, IQR 3.46-6.03), respectively. The difference in the antibody response between the dry and wet season was statistically significant (p < 0.001).

#### Antibody prevalence and the age group

The prevalence of antibody response to MSP2, EBA-175 and PfRh2a in different age groups is shown in Table 3. The antibody prevalence to MSP2 and EBA-175 increased in the older age groups, whereas for PfRh2a, the antibody prevalence is relatively constant by age.

**Table 3:** **IgG prevalence to MSP2, EBA-175 and PfRh2a** ***Plasmodium falciparum*** **based on age group**

Age groups	IgG MSP2 (unit)		IgG EBA-175 (unit)		IgG PfRh2a (unit)	
	Antibody prevalence (%)	Median (IQR)	Antibody prevalence (%)	Median (IQR)	Antibody prevalence (%)	Median (IQR)
0-12 years	31.82%	1.07	32.73%	3.61	36.36%	1.49
(n = 110)		(0.40-2.76)		(1.97-7.47)		(0.78-2.78)
13-40 years	37.30%	1.41	38.10%	3.84	33.33%	1.49
(n = 126)		(0.74-3.78)		(2.44-9.27)		(1.01-3.05)
>40 years	42.86%	1.74	40.82%	3.38	36.73%	1.49
(n = 49)		(0.40-4.29)		(2.21-8.59)		(0.78-2.78)
p (ROH) (Kruskall Wallis test)		0.15		0.31		0.66
p (Spearman Correlation)		0.07		0.31		0.97

#### Antibody level and the age groups

Based on the age group, the median levels of IgG response to MSP2 and EBA-175 were slightly higher in subjects ≥12 years old *versus* those <12 years old (Table 4). The difference in the level of antibodies, however, was only statistically significant for the MSP2 (p = 0.05). The median level of antibody to PfRh2a in all age groups was relatively constant.

**Table 4:** **Level of IgG MSP2, EBA-175 and PfRh2a (units) in age < = 12 years and age >12 years groups (n = 285)**

Variables	Age < =12 years (n = 110)		Age >12 years (n = 175)		P*
	Median	IQR	Median	IQR	
IgG MSP2 (unit)	1.07	(0.40-2.76)	1.41	(0.74-4.12)	0.05
IgG EBA-175 (unit)	3.61	(1.98-7.48)	3.84	(2.45-8.82)	0.16
IgG PfRh2a (unit)	1.50	(0.78-2.77)	1.50	(0.78-2.89)	0.68

(*) was determined by using Mann–Whitney test.

#### Antibody level and malaria positivity

Of the 497 subjects included in this study, 59 were confirmed to be falciparum infected, based on the PCR detection method (Table 5). The median antibody level to MSP2, EBA-175 and PfRh2a antigens was higher in the falciparum-infected subjects *versus* uninfected subjects. The difference in antibody level to MSP2, EBA-175 and PfRh2a is not statistically significant.

**Table 5:** **Antibody level to MSP2, EBA-175, PfRh2a and malaria positivity**

Variables	***P. falciparum***-positive (n = 59)		***P. falciparum***-negative (n = 438)		P*
	Median	IQR	Median	IQR	
IgG MSP2 (unit)	2.42	(0.74-10.15)	1.74	(0.74-4.47)	0.11
IgG EBA-175 (unit)	6.58	(1.98-14.57)	4.30	(2.45-10.16)	0.42
IgG PfRh2a (unit)	2.51	(0.78-4.77)	1.50	(0.78-3.89)	0.31

(*) was determined by using Mann–Whitney test.

## Discussion

The antibody responses to the *P. falciparum* merozoite proteins MSP2, EBA-175 and PfRh2a were detected in subjects living in the malaria-endemic areas of Mamuju District, West Sulawesi Province, Indonesia and the responses are significantly higher in the wet season. The findings indicate that the three antigens might potentially be used as markers for exposure to malaria. Antibody responses to various merozoite proteins have been well documented in different population studies, but only few of the antigens was found to confer protection against clinical malaria [16]. In Indonesia, there are very limited data regarding the antibody profiles of merozoite proteins and their association with protection to malaria [17–19]. A previous study showed that antibody response to MSP1 and apical membrane antigen 1(AMA-1) is higher in high transmission season in comparison to the low transmission season. The findings are in accordance with the results of this study where the antibody response to the three merozoite antigens examined was found to be higher in the wet season when the transmission was high [10, 20]. In a cohort study in Gambian children, increased levels of serum IgG antibodies against *P. falciparum* merozoite antigens were observed in many children during the rainy season. The levels of antibody to AMA-1, EBA-175, MSP1_19_ and MSP2 increased at the end of the rainy season in children who had experienced clinical symptoms of malaria [21].

Antibody responses to MSP2 and EBA-175 have also been associated with protection from clinical malaria in adult subjects [22]. In this study, although the antibody levels in older age groups were higher for both MSP2 and EBA-175, the differences were only statistically significant for MSP2 due to the high variation within the age group. The levels of antibody response to MSP2, EBA-175 and PfRh2a were also found to be slightly higher in *P. falciparum*-positive subjects although this difference was not statistically significant. Further study involving larger sample size and specific IgG subclasses may provide a more conclusive result in this regard.

Antibody response to the PfRh2a antigen was also observed, and the levels tend to constant with age. PfRh2a and PfRh2b proteins are identical for 2700 N-terminal amino acids and differed only in a C-terminal 500 amino acid region, which includes a unique ectodomain, transmembrane domain and cytoplasmic domain [5]. Antibodies raised against the binding domains of the PfRh2a and PfRh2b at the N terminus blocked the merozoite invasion [9]. It is therefore possible that the antibody response to PfRh2a detected in this study, may have simultaneously been targeted to PfRh2b antigen.

A study involving 206 children in Papua New Guinea demonstrated that the IgG1 and IgG3 levels were predominantly higher in children who had parasitaemia detected by PCR than those who did not have malaria. Interestingly, they found that antibody responses to PfRh2a were associated with age and active infection. High levels of IgG to PfRh2 were strongly associated with protection from malaria symptoms and parasitaemia [15].

## Conclusion

Antibody responses against three merozoites proteins were observed in the population of Mamuju District, West Sulawesi Province, Indonesia. The increase of the antibody response during the wet season indicates the high malaria transmission during the period. Therefore, MSP2, EBA-175 and PfRh2a are potentially useful as markers for malaria exposure. Among the three merozoite proteins examined, only the MSP2 showed protective efficacy as indicated in the higher level in the older age group and malaria-positive subjects. This study has a limitation as the assays were performed using archived filter-paper blood spots collected during the baseline malaria survey. Analysis of the total samples collected during the survey, although it would be laborious and require more logistics, may provide more conclusive results.
